# Enhancing Neuronal Networks with *Rhinella schneideri* Skin Secretion Molecules: Implications for Neurodegenerative Disorders

**DOI:** 10.3390/toxins18060271

**Published:** 2026-06-20

**Authors:** Giovanna Arruda Caires, Isabela Souza Pereira, Carlos DeOcesano-Pereira, Daniel Carvalho Pimenta, Irina Kerkis, Juliana Mozer Sciani, Hugo Vigerelli

**Affiliations:** 1 Programa de Pós-Graduação em Ciências-Toxinologia do Instituto Butantan, Sao Paulo 05503-900, Brazil; 2 Laboratory of Genetics, Butantan Institute, Sao Paulo 05503-900, Brazil; 3 Center of Excellence in New Target Discovery, Butantan Institute, Sao Paulo 05503-900, Brazil; 4 Laboratory of Biochemistry and Biophysics, Butantan Institute, Sao Paulo 05503-900, Brazil; 5 Laboratory of Ecology and Evolution, Butantan Institute, Sao Paulo 05503-900, Brazil; 6 Laboratory of Natural Products, São Francisco University, Bragança Paulista 12916-900, Brazil

**Keywords:** neurodegenerative diseases, *Rhinella schneideri*, skin secretion

## Abstract

Neurodegenerative disorders, including Parkinson’s and Alzheimer’s diseases, are hallmarked by the progressive degeneration of neuronal networks. Given the lack of disease-modifying cures, current therapies are limited to symptomatic relief. Here, we investigated the neurotrophic potential of the skin secretion (SS) from *Rhinella schneideri*, its polar fraction (PF) and nonpolar (NPF) fraction, and respective subfractions on the morphology of neuron-like cells. Following initial H_2_O-CH_2_CL_2_ partitioning, PF and NPF subfractions were isolated via RP-HPLC. Chemical characterization using LC-MS-IT-TOF identified eight distinct molecules, notably bufotenine and marinobufagin. Cytotoxicity screening established safe working concentrations (100–250 ng/mL for SS/PF; 250–500 ng/mL for NPF and subfractions) for downstream morphological evaluations using High Content Screening (HCS). The subfraction polar 5 (SfP5) elicited a robust neurotrophic response, significantly enhancing all assessed morphometric parameters: total neurite outgrowth (+72%), branching points (+120%), maximum process length (+60%), and total number of processes (+35%). Our data show that *Rhinella schneideri* SS contains molecules that improve in vitro neuronal networks, serving as a promising source for preliminary screening of neuroprotective effects.

## 1. Introduction

Neurodegenerative disorders, such as Alzheimer’s, Parkinson’s, amyotrophic lateral sclerosis, and frontotemporal lobar dementia, account for over 6.8 million deaths worldwide (1). In most neurodegenerative disorders, slow, progressive loss of neurons and a reduction in dendritic spine density occur. This loss of connectivity results in impaired synaptic transmission and, consequently, neuronal death, alongside other features driving degeneration. In Alzheimer’s disease (AD), for example, beta-amyloid peptides in the synaptic cleft hinder communication between neurons, leading to the loss of dendritic spines (2). Currently, these diseases have no cure. A potential strategy to slow neuronal loss is to stimulate the formation of neuronal networks, thereby improving communication between healthy neurons and those already undergoing neurodegeneration.

In recent years, the pharmaceutical industry has produced hundreds of medications that have increased life expectancy. However, the high production costs and limited molecular targets of these synthetic drugs have decreased the pharmaceutical sector’s interest in them. In this context, researchers have increasingly turned to natural products for the discovery of new medications [1,2].

Animal toxins and skin secretions serve as rich sources of promising bioactive molecules for new discoveries and act as valuable biological tools for identifying molecular targets involved in various diseases. Despite the pharmaceutical industry’s historical reluctance toward animal venoms, their application in alternative and traditional medicine has a long history. Chan Su, for instance, is a preparation derived from the dried skin extract of toads, used in traditional Chinese medicine for over a millennium as a diuretic, anesthetic, and anticancer agent [3]. Furthermore, snake venoms have been utilized since the 1930s to treat conditions such as asthma, polio, multiple sclerosis, rheumatism, and severe pain [4].

Literature demonstrates that molecules found in amphibian venoms can act as antitumor [5,6], analgesics [7], antiparasitic [8] and anti-inflammatory agents [9]. They may also directly impact the treatment of diseases, such as diabetes [10], heart failure [11], neuropsychiatric diseases [12] and rabies [13,14].

Venom composition exhibits both inter- and intra-specific variations driven by biological and environmental factors, including age, climate, diet, and adaptive evolution [15,16]. The chemical profile of toad venoms is highly complex and can be broadly divided into two groups of low molecular weight molecules: biogenic amines (e.g., adrenaline, noradrenaline, bufotenines) and steroidal derivatives (e.g., bufotoxins and bufadienolides). Adrenaline and noradrenaline act as agonists of the sympathetic autonomic nervous system, inducing cutaneous and visceral vasoconstriction, muscular vasodilation, bronchodilation, and increased heart rate and contractility. Bufotenines exert hallucinogenic effects on the central nervous system, while bufotoxins and bufodienolides display cardiotoxic activity through the inhibition of Na^+^/K^+^/ATPase pumps [17].

However, from a pharmacological perspective, the biological outcome of these molecules, whether toxic, psychoactive, or therapeutic, is heavily dose-dependent and structure-specific. By fractionating the crude secretion, it becomes possible to bypass the classical toxicities and isolate specific molecular scaffolds for targeted therapies. Although the exact neurotrophic mechanisms of these specific fractions remain largely unexplored, hypotheses can be drawn based on their chemical nature. The polar fraction, rich in alkaloid derivatives such as bufotenine, shares significant structural homology with the neurotransmitter serotonin [18]. Consequently, its possible mechanism of action may involve interaction with serotonergic receptors or other monoaminergic pathways known to modulate neuroplasticity and neurite outgrowth [19]. Conversely, the nonpolar fraction is predominantly composed of steroidal derivatives, such as bufadienolides, which are classic modulators of Na^+^/K^+^/ATPase pumps [20,21]. While high concentrations of these steroids induce toxicity, sub-lethal doses could potentially trigger downstream intracellular signaling cascades, such as localized calcium transients, that favor cytoskeletal reorganization and neuronal network expansion. Furthermore, bufotenine, an alkaloid present in the skin secretion of toads from the genus Rhinella, has been demonstrated as a promising molecule for the treatment of rabies, a disease that targets the central nervous system. This therapeutic potential is attributed to its ability to delay the onset and reduce the severity of rabies-induced symptoms in mice, increasing survival by 40%. In addition, it inhibited rabies virus infection in N2A neuroblastoma cells [13,14].

So, in this study, bioactive molecules from *Rhinella schneideri* Skin Secretion (SS) were investigated in neuron-like cells in the neuronal network.

## 2. Results

### 2.1. The Liquid–Liquid Partition Is Capable of Concentrating the Polar and Nonpolar Molecules from Total Skin Secretion

**Figure 1:**
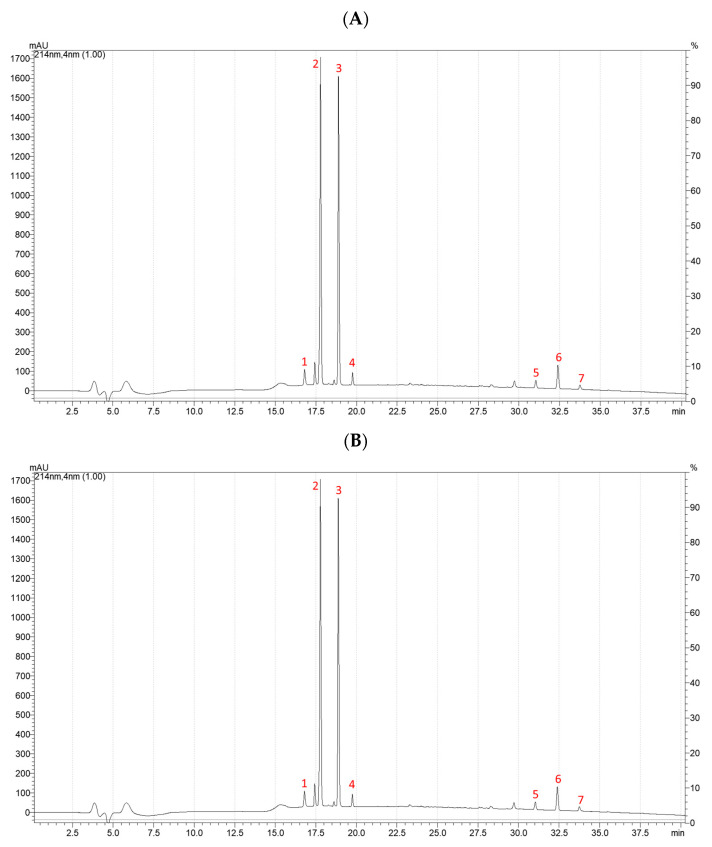

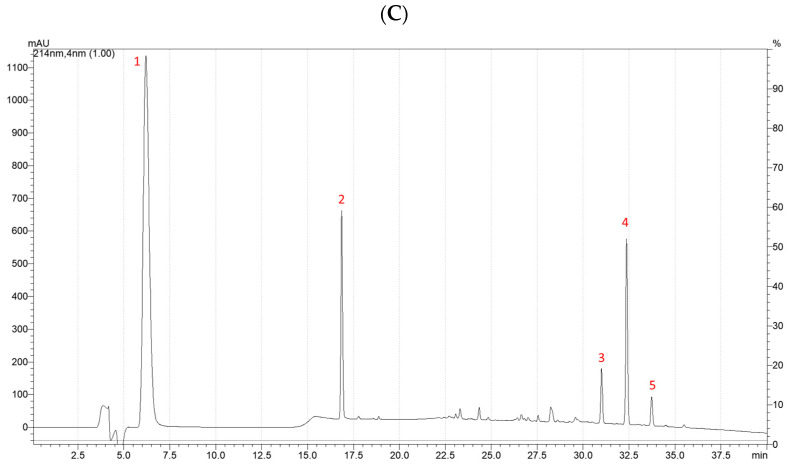
C18-RP-HPLC chromatographic profiles (*λ* = 214 nm). Water partition SS chromatographic profile (**A**). Polar fraction (PF) chromatographic profile (**B**). Non-polar fraction (NPF) chromatographic profile (**C**). The red numbers represent the collected fractions.

Polar fraction (PF) and non-polar fraction (NPF) were obtained by H_2_O-CH_2_CL_2_ partition. PF and NPF subfractions were collected using Reverse-Phase High-Performance Liquid Chromatography (RP-HPLC), as shown in Figure 1A–C. Seven subfractions from the PF and 5 from NPF were obtained, according to the numbers depicted in the chromatograms.

**Figure 2:**
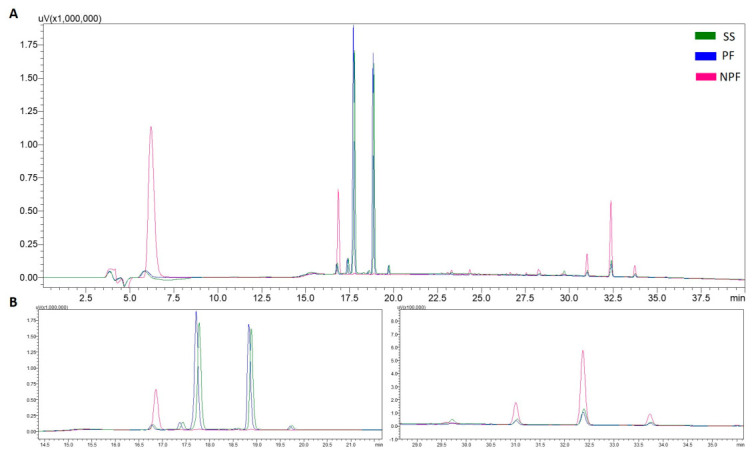
Overlaid C18-RP-HPLC chromatographic profiles (*λ* = 214 nm). The curves represent the profiles of SS (green), PF (blue), and NPF (pink). Full chromatograms (**A**). 14.5–21 and 29–35 min zoom (**B**).

PF and NPF chromatographic profiles were obtained by RP-HPLC to confirm the liquid–liquid partition SS. The overlay of the SS, PF and NPF chromatographic profiles, shown in Figure 2, indicates that the liquid–liquid technique effectively concentrated the polar and non-polar molecules.

### 2.2. Molecules Identified in the SS by Mass Spectrometry

The fractions collected from the SS (Figure 1A) during the chromatographic process were subjected to mass spectrometry analysis (Supplementary Figure S1). Based on their *m*/*z* values and comparisons with the existing literature, eight compounds were putatively identified, as summarized in Table 1.

### 2.3. Cytotoxic Assay

**Figure 3:**
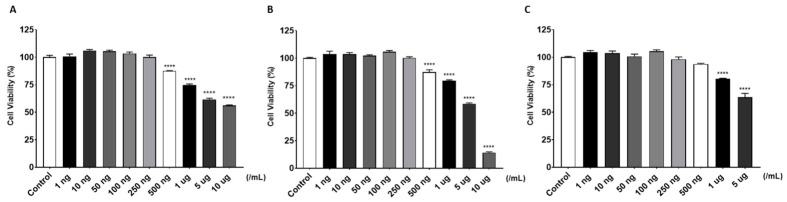
Neuron-like viability after 24 h of exposure to SS (**A**), PF (**B**), and NPF (**C**). The control group received only DMEM/F12 with 10% FBS (Fetal Bovine Serum) and 10 µM retinoic acid. All values are the results of three independent experiments with three replicates and are expressed as mean + SEM; **** *p* ≤ 0.0001 vs. control. (ANOVA and Tukey’s post-test).

To identify the optimal non-cytotoxic working concentrations, an initial dose–response screening was performed, evaluating a broad range of concentrations from 1 ng/mL to 10 µg/mL. A concentration-dependent decrease in neuron-like cell viability was observed following a 24 h exposure to SS, PF, and NPF across the tested range (Figure 3A, B, and C, respectively). A significant reduction in cell viability was observed starting at 500 ng/mL for SS and PF, and at 1 μg/mL for NPF. Consequently, the highest non-cytotoxic concentrations were selected for the subsequent experiments: 100 and 250 ng/mL for both SS and PF, and 250 and 500 ng/mL for NPF.

**Figure 4:**
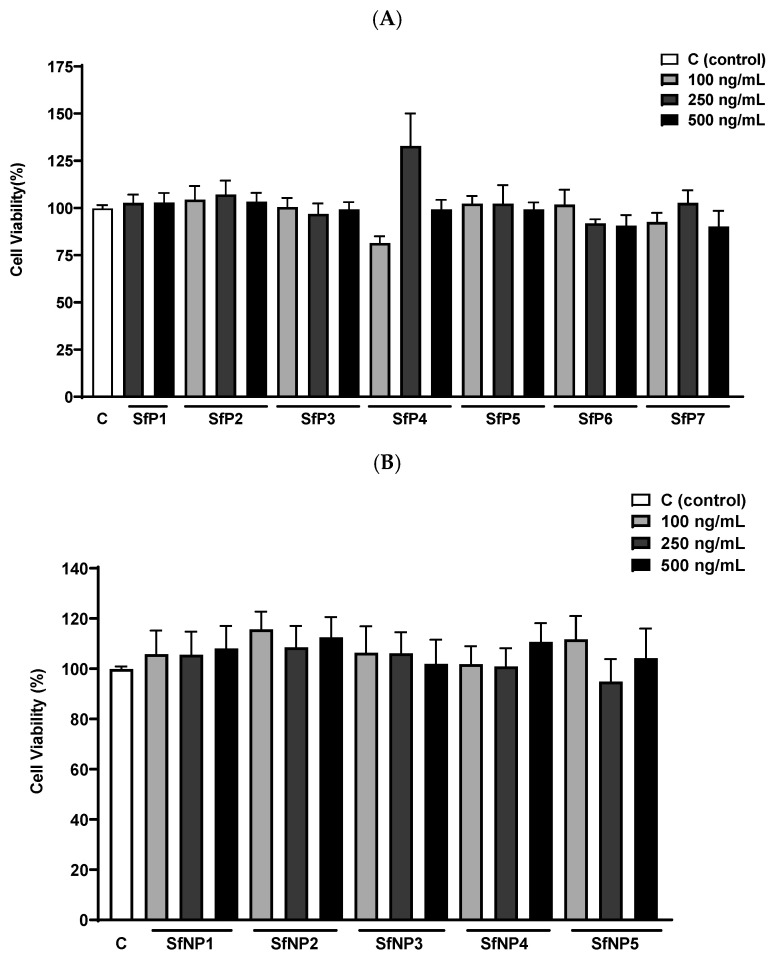
Neuron-like viability after 24 h of exposure to the SfP (**A**) and SfNP (**B**). The control group received only DMEM/F12 with FBS 10% and 10 µM retinoic acid. All values are the results of three independent experiments with three replicates and are expressed as mean + SEM; (ANOVA and Tukey’s *post hoc* test).

Moreover, none of the tested concentrations (100, 250, and 500 ng/mL) of the Polar SubFractions (SfP) and NonPolar Subfractions (SfNP) significantly reduced neuron-like cell viability after 24 h of incubation (Figure 4). Therefore, the concentrations of 250 and 500 ng/mL were selected for the subsequent assays.

### 2.4. SfP5 Is Capable of Improving the Analysis Parameters of the Neuronal Network in Neuron-like Cells

**Figure 5:**
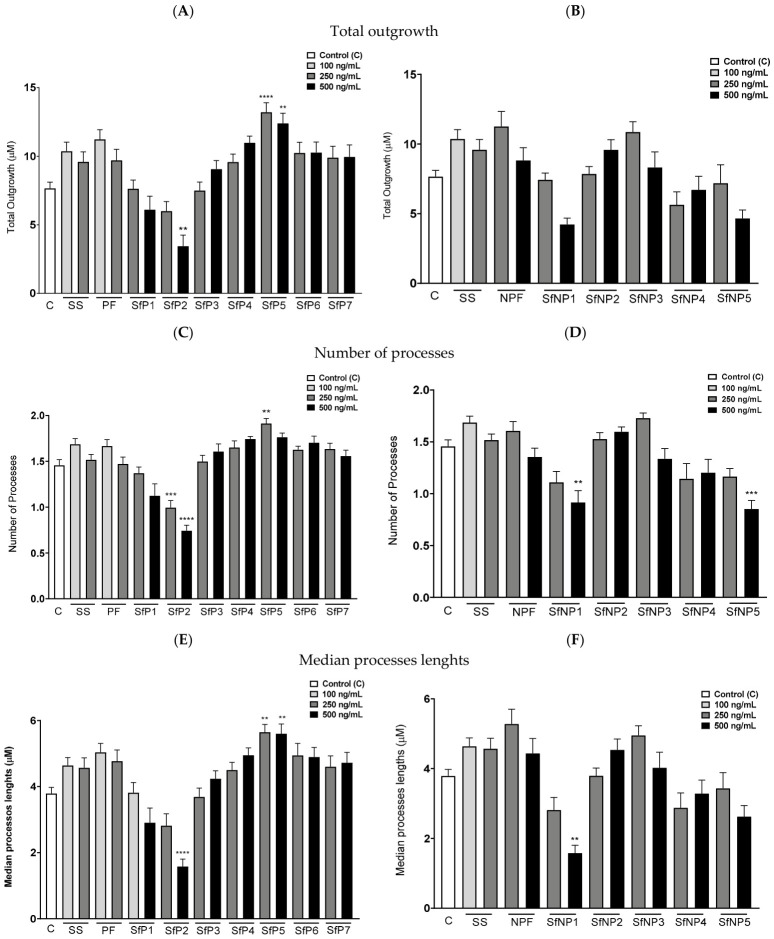

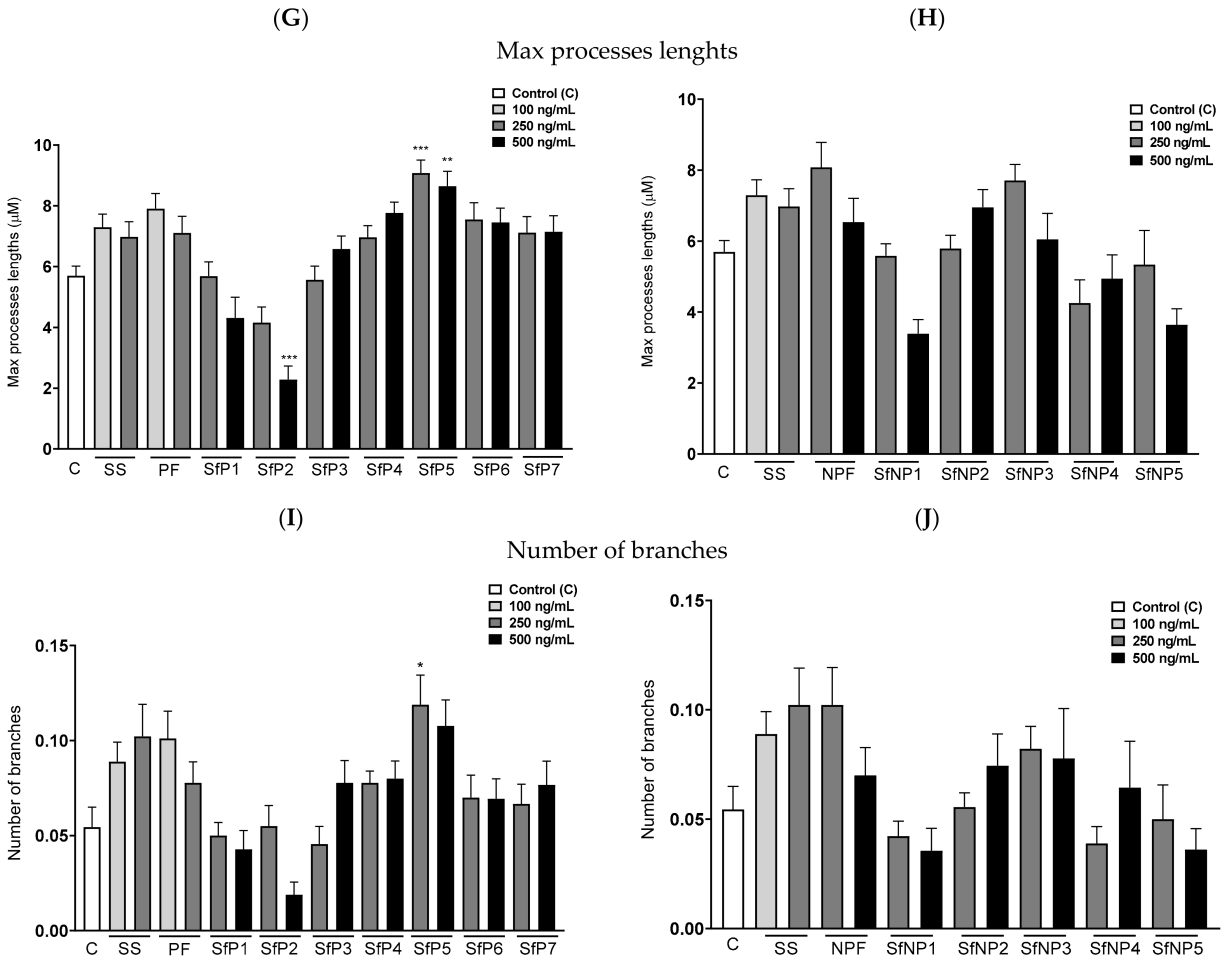
Effect of *Rhinella schneideri* SS, PF, NPF and subfractions on the morphology of neuron-like cells. Neuron-like cells were stimulated with different concentrations of SS and PF (100 or 250 ng/mL), NPF, SfP 1–7 and SfNP 1–5 (250 or 500 ng/mL), or with DMEM/F12 supplemented with 1% FBS and 0.1% antibiotic + retinoic acid (10 µM) (C = control), for 24 h. Neurite outgrowth was assessed using a High-Content Screening (HCS) assay following immunostaining for β III tubulin. Parameters used in morphological endpoints included total outgrowth (**A**,**B**), number of processes (**C**,**D**), neurite length (**E**–**H**), and neurite branch (**I**,**J**) points for each identified cell, which were quantified using the automated image analysis features of the MetaXpress High-Content Image Acquisition & Analysis Software (Molecular Devices, version 6.2.3.733). On the left are subfractions from PF, and on the right are subfractions from NPF. All values are the results of three independent experiments with three replicates and are expressed as mean + SEM. Data were analyzed using one-way ANOVA followed by Tukey’s *post hoc* test; *p* ≤ 0.05 (*), *p* ≤ 0.01 (**), *p* ≤ 0.001 (***), *p* ≤ 0.0001 (****) vs. control.

To investigate potential neurotrophic effects, the neuronal network morphology was analyzed. Neuron-like cells were stimulated for 24 h with 100, 250, or 500 ng/mL of SS, PF, NPF, and subfractions. After this period, the cells were stained with anti-βIII tubulin, followed by Alexa 488-conjugated secondary antibody to detect cell bodies and neurites in neuron-like cells by High Content Screening assay. The graphics show (Figure 5) that the SfP5 could statistically improve all the parameters below. On the other hand, no significant improvement was detected when subfractions from NPF were used. The total outgrowth represents the total length of skeletonized outgrowth in µm (corrected for diagonal lengths); the number of processes is the number of outgrowths that are connected to cell bodies; median/max process length are the median/max value of the outgrowth lengths (in μm) associated with the cell’s various processes, and the neurite branches are the number of branching junctions of all the processes connected to the cell body.

**Figure 6:**
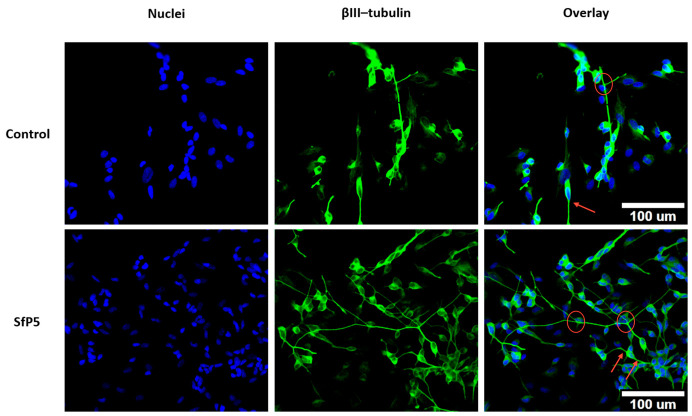
Representative High Content Screening images demonstrating the improvement of neuronal network parameters by SfP5. Neuron-like cells were stimulated with 250 ng/mL of SfP5, or with DMEM/F12 supplemented with 1% FBS, 0.1% antibiotic, and 10 µM retinoic acid (Control group), for 24 h. After fixing and staining with specific antibodies, images were captured using the ImageXpress Micro Confocal High Content Imaging System (Molecular Devices). Nuclei: blue, βIII- tubulin: green. Red circles indicate ramifications, and red arrows trace the neurite processes. Magnification: 20× (scale bar: 100 µm).

Since the initial screening revealed that the SfP5 subfraction was capable of significantly enhancing all analyzed parameters of the neuronal network, this specific fraction was selected for an in-depth morphological representation. Figure 6 represents the total outgrowth, number of processes, median/max process length, and neurite branches in neuron-like cells stimulated with 250 ng/mL of SfP5 compared to the control group, showing the neuron’s connection improvement after treatment. Some representative images illustrating the quantified and described parameters are presented below (Figure 6).

### 2.5. Chemical Profiling of the Active SfP5 Fraction

Given the robust morphological protection observed in vitro, we sought to perform an initial chemical characterization of the active SfP5 fraction. Full-scan mass spectrometry (MS1) profiling revealed three prominent precursor ions at *m*/*z* 304, 330, and 403. To expand this preliminary characterization, tandem mass spectrometry (MS/MS) was performed on these major signals (Supplementary Figure S2). The precursor ion at *m*/*z* 330 generated a major product ion at *m*/*z* 304, together with additional fragments at *m*/*z* 182 and 150. Interestingly, direct fragmentation of the *m*/*z* 304 precursor yielded the same dominant ions, with *m*/*z* 182 representing the base peak in both spectra. The marked similarity between the fragmentation profiles, combined with the formation of *m*/*z* 304 from the *m*/*z* 330 precursor, strongly suggests that these ions correspond to structurally related molecular species sharing a common molecular scaffold and fragmentation pathway. Finally, the ion at *m*/*z* 403 remained stable and did not yield detectable fragments under the applied collision energy conditions.

## 3. Discussion

The skin secretion of toads from the Bufonidae family is mainly composed of alkaloid molecules (polar-hydrophilic) and steroidal derivatives (nonpolar-hydrophobic) [17]. Because polar molecules are hydrophilic, the blue peaks (FP) in Figure 2A were more intense at the beginning of the gradient, when the main component of the mobile phase was an aqueous solution. In contrast, the pink peaks (FA) were more intense when the organic solvent (acetonitrile) approached 100%, reflecting their more hydrophobic nature. Comparing the chromatographic profiles, it is evident that some subfractions are common to both the PF and NPFs (Figure 2A, B). Despite differences in peak intensity, they exhibit identical retention times. This overlap is observed between SfP2 and SfNP2, as well as among SfP5, 6 and 7, which superpose with SfNP3, 4, and 5. Due to their amphipathic nature, these subfractions may contain the same molecules that were not completely separated during the liquid–liquid partition. For instance, marinobufagin and bufalin are steroidal compounds that possess some solubility in aqueous media, as estimated from their molecular structures [24]. All molecules putatively identified in this study, except those with *m*/*z* 358 and *m*/*z* 405, have already been described in the literature as components of the parotid gland secretion of *Rhinella schneideri*. It is important to clarify that these unannotated peaks are not being claimed as novel compounds, but simply represent molecules that could not be tentatively identified at this time. The putatively annotated compounds include bufotenine, dehydrobufotenine, marinobufagin, telocinobufagin, bufalin [21], adipoyl arginine, bufotenidine, and suberoyl arginine [19].

As demonstrated herein, the SfP5 subfraction improved the morphological parameters of neuron-like cells. Specifically, the 250 ng/mL concentration successfully increased all evaluated parameters, promoting cytoskeleton elongation and increasing the number of processes, branching points, and overall neurite length. Meanwhile, the 500 ng/mL concentration also enhanced cytoskeleton elongation and overall neurite length, but did not significantly increase the number of branches or processes. These data are relevant to neurodegenerative diseases such as AD. The accumulation of amyloid plaques between neurons impairs synaptic transmission, activates autophagic and mitochondrial apoptotic pathways, and triggers the release of inflammatory mediators. Together, these events drive neurodegeneration, which initially manifests as neurite dystrophy. Basal action potential transmission coupled with high synaptic activity increases the number of postsynaptic ionotropic glutamate receptors, such as AMPA receptors, thereby inducing dendritic spine growth and potentiating synaptic transmission. In neurons, synaptic stimulation tightly regulates the density of postsynaptic AMPA receptors. Consequently, low levels of synaptic stimulation can lead to the removal of these receptors and a subsequent reduction in dendritic spine growth. In AD, for example, the presence of amyloid plaques impairs synaptic transmission, resulting in the endocytosis of AMPA receptors and leading to dendritic spine loss [25,26,27,28,29]. Moreover, considering the dysregulation of the cholinergic pathway in AD, another potential mechanism for the SS molecules involves their participation in acetylcholine-mediated neurite outgrowth, a hypothesis that warrants further investigation [30]. Therefore, expanding and restoring the neuronal network could serve as a viable alternative to counteract the cellular communication damage caused by AD.

Comparing the chromatographic profiles of SS, PF, and NPF, it can be observed that the peak corresponding to SfP5 is constant for all profiles, having the same retention time and being represented in fraction 5 of SS and SfNP3. The direct analysis of the active subfraction SfP5, using full-scan mass spectrometry (MS1), revealed three prominent precursor ions at *m*/*z* 304, 330, and 403. The signal at *m*/*z* 403, which was also detected in the mass spectrometry analysis of peak 5 from the crude SS, putatively corresponds to telocinobufagin, a steroid common in the skin secretion of toads. Our tandem mass spectrometry (MS/MS) SfP5 analysis further supported this, as the *m*/*z* 403 ion remained stable and did not yield detectable fragments under the applied collision energy, a characteristic of rigid steroidal scaffolds.

Furthermore, the MS/MS analysis of the remaining prominent ions revealed a clear precursor–product relationship between the signals detected at *m*/*z* 330 and 304. Fragmentation of the *m*/*z* 330 precursor generated a major product ion at *m*/*z* 304, while both precursor ions subsequently produced highly similar product-ion spectra dominated by fragments at *m*/*z* 182 and 150. The shared fragmentation behavior and the common base peak at *m*/*z* 182 strongly suggest that these ions represent structurally related molecular species containing the same core scaffold. Therefore, rather than representing independent constituents, the signals at *m*/*z* 330 and 304 may correspond to different molecular forms of the same metabolite or to closely related analogs co-eluting within the SfP5 fraction. As a steroid, telocinobufagin should be more abundant in the dichloromethane fraction compared to the water fraction. However, SfNP3, which would correspond to this same molecule, could not induce the increase in morphological parameters of neuron-like cells. Therefore, the molecule responsible for SfP5’s effect may not be telocinobufagin acting alone. Instead, the biological activity could be primarily associated with the structurally related compounds represented by the ions at *m*/*z* 330 and 304. Alternatively, the observed morphological protection could result from a synergistic interaction between telocinobufagin (or a more polar analog thereof) and these co-eluting constituents. None of the nonpolar subfractions, nor the total nonpolar fraction, significantly increased the analyzed parameters.

## 4. Conclusions

In conclusion, the present study demonstrates that low-molecular-weight molecules from *Rhinella schneideri* skin secretion effectively enhance the neuronal network morphology in neuron-like cells. This highlights their promising potential as in vitro tools for investigating neurodegenerative diseases, deciphering pathophysiological mechanisms, and driving the development of novel therapeutics.

## 5. Materials and Methods

### 5.1. Fractionation of Rhinella schneideri Skin Secretion by HPLC

The lyophilized *Rhinella schneideri* skin secretion was donated by the Laboratory of Cell Biology, Butantan Institute, São Paulo, SP, Brazil. A liquid–liquid partition (water: dichloromethane) was performed to obtain concentrated fractions of polar and nonpolar molecules. From the total SS sample, 10 mg were separated and dissolved in 5 mL of ultrapure water and 5 mL of dichloromethane. After 24 h, the aqueous partition was collected and lyophilized, and the organic partition was left in a fume hood until complete evaporation of the organic solvent. The SS, PF, and NPF were fractionated, and the resulting subfractions were collected using RP-HPLC in a binary HPLC system (20A Prominence, Shimadzu Co., Kyoto, Japan). 50 µL of the samples were injected into a C18 column (ACE 250 × 4.6 mm), with solvents (A) trifluoroacetic acid/H_2_O (1:1000) and (B) trifluoroacetic acid/acetonitrile/H_2_O (1:900:100) at a constant flow rate of 1 mL/min at 40 °C. The gradient was from 0 to 100% of solvent B in 35 min. Fractions were manually collected and submitted to mass spectrometry (MS ESI-IT-TOF) analyses.

### 5.2. Mass Spectrometry

The fractions collected from the SS, as well as the active subfractions, were subjected to drying (GeneVacSF50) and subsequently dissolved in a solution of 50% acetonitrile and water containing 0.5% formic acid for MS and/or MS2 analyses. The analyses were performed using an ESI-IT-TOF mass spectrometer system (Shimadzu Co., Kyoto, Japan). Three microliters of the samples were injected using an autoinjector in positive ionization mode with a constant flow rate of 50 μL/min. The interface voltage was set at 4.5 kV, and the detector voltage was 1.76 kV, with an interface temperature of 200 °C. For the MS2 analyses, the most intense precursor ions were automatically selected for fragmentation using argon as the collision gas with 50% collision energy. The mass spectra were acquired over a range of 50 to 2000 *m*/*z*. All obtained data were processed and analyzed using the LCMS solution software v 3.0 (Shimadzu Co., Japan).

### 5.3. Cell Culture and Differentiation Protocol for Neuron-like Cells

SH-SY5Y human neuroblastoma cells obtained from the European Collection of Authenticated Cell Culture (ECACC, Salisbury, Wiltshire, UK, #94030304) were maintained in a mixture of 1:1 Ham’s F12 and Dulbecco’s Modified Eagle Medium (DMEM) supplemented with 10% Fetal Bovine Serum (FBS) and antibiotic streptomycin/penicillin 1%, in a humidified atmosphere of 5% of CO_2_ in air at 37 °C. The cell medium was replaced every 3 days until confluence. For differentiation into neuron-like cells, using a protocol adapted from Lopes et al. [31], the cells were plated in a 96-well plate at a density of 1 × 10^4^ cells per well. After 24 h of cell plating, differentiation was induced by lowering the FBS in the culture medium to 1% and adding retinoic acid at 10 µM. This treatment medium was replaced after 3 days, and the experiment was conducted over an 8-day period.

### 5.4. Cell Viability

The Cell Count Kit-8 (CCK-8, Dojindo Molecular Technologies, Rockville, MD, USA) assessed the viability of neuron-like cells. After differentiation into neuron-like cells, the cells were incubated, either untreated (Control: DMEM/F12 medium with 1% FBS, 0.1% penicillin/streptomycin and 10 µM retinoic acid) or with the following concentrations of SS, FP, or NFP: 1 ng/mL, 10 ng/mL, 50 ng/mL, 100 ng/mL, 250 ng/mL, 500 ng/mL, 1 µg/mL, 5 µg/mL, or 10 µg/mL. Additionally, the cells were treated with subfractions collected at concentrations of 100 ng/mL, 250 ng/mL, or 500 ng/mL for 24 h. The CCK-8 solution was added at a ratio of 1:20 in each well for 4 h, and the absorbance value of each well was measured at 450 nm using a spectrophotometer. Cell viability was calculated as the percentage of the sample’s absorbance relative to the control (100%).

### 5.5. Neuron-like Cells Morphological Analysis by HCS

For the High-Content Screening assay, cells were seeded at 1 × 10^4^ per well into black Advanced TC 96-well microplates (Greiner Bio-One, Frickenhausen, Germany, #655986). On the 8th day of differentiation, the cells received the following treatment. The control group, DMEM/F12 with 1% FBS and 10 µM retinoic acid, and the treatment group had different SS, PF, NPF, and subfractions concentrations for 24 h. To detect cell bodies and neurites in neuron-like cells, at room temperature, cells were fixed with 100 μL of 4% paraformaldehyde for 1 h. After this, they were permeabilized with Triton-X 100 for 5 min and washed with PBS. So, the cells were blocked with bovine serum albumin 1% for 30 min at 4 °C and incubated with the primary antibody (Mouse anti-βIII-tubulin human, #ab78078, Abcam, Cambridge, UK); this stage was performed overnight at 4 °C. After washing the cells with PBS, they were incubated with a secondary antibody (Goat anti-mouse Alexa Fluor 488, Invitrogen, Thermo Fisher Scientific, Waltham, MA, USA, #A-10680, 1:1000) for 1 h at room temperature. In the last phase, the DAPI (Sigma-Aldrich, Burlington, MA, USA, #D9564, 1:1000) was added to stain the nuclei. HCS was performed on the plates using MetaXpress High-Content Image Acquisition & Analysis Software (Molecular Devices, Sunnyvale, CA, USA; version 6.2.3.733). Images were automatically captured using the ImageXpress Micro Confocal High Content Imaging System (Molecular Devices, Sunnyvale, CA, USA). The resulting images were analyzed using the same software version for quantification. Parameters used in morphological endpoints included total outgrowth, number of processes, neurite length, and neurite branch points for each identified cell. A total of 16 images per well were captured at 20× magnification. Subsequent quantification and correlation of neuron and neurite morphology were performed using an automated HCS assay.

### 5.6. Statistical Analysis

All quantitative data are expressed as the mean + standard error of the mean (S.E.M.). Data were analyzed by one-way ANOVA followed by Tukey’s *post hoc* test for multiple comparisons. The normality and homoscedasticity of all samples were confirmed prior to analysis. All statistical analyses were performed using GraphPad Prism version 8.0.1 (GraphPad Software, San Diego, CA, USA). The level of statistical significance was set at *p* < 0.05. 

## Figures and Tables

**Table 1:** Identified molecules from *Rhinella schneideri* skin secretion.

Fraction	*m*/*z*	Tentative Identification	Reference
1	303 *m*/*z*	Adipoyl arginine	PETROSELLI et al., 2018 [22]
2	205 *m*/*z* and 219 *m*/*z*	Bufotenine (205 *m*/*z*) and bufotenidine (219 *m*/*z*)	SCHMEDA-HIRSCHMANN et al., 2014 [23]
3	203 *m*/*z* and 405 *m*/*z*	Dehydrobufotenine (203 *m*/*z*) and 405 *m*/*z* are not identified in the literature	SCHMEDA-HIRSCHMANN et al., 2014 [23]
4	331 *m*/*z*	Suberoyl arginine	PETROSELLI et al., 2018 [22]
5	403 *m*/*z* and 425 *m*/*z*	Telocinobufagin and its adduct	PETROSELLI et al., 2018 [22]
6	401 *m*/*z* and 423 *m*/*z*	Marinobufagin and its adduct	PETROSELLI et al., 2018 [22]
7	387 *m*/*z* and 358 *m*/*z*	Bufalin and 358 *m*/*z* are not identified in the literature	SCHMEDA-HIRSCHMANN et al., 2014 [23]

## Data Availability

The original contributions presented in this study are included in the article. Further inquiries can be directed to the corresponding authors.

## Supplementary Materials

The following supporting information can be downloaded at: https://www.mdpi.com/article/10.3390/toxins18060271/s1, Figure S1: The numbers indicated above the peaks correspond to the compounds detailed in Table 1. Mass spectrometry analysis of the crude skin secretion (SS). Representative mass spectrometry (MS) spectra of the major peaks collected from the crude skin secretion of *Rhinella schneideri*. The numbers indicated above the signals (Peak 1, Peak 2, etc.) correspond to the specific peaks and their respective putatively identified compounds previously detailed in Table 1. Figure S2: Mass spectrometry (MS) and tandem mass spectrometry (MS/MS) characterization of the biologically active SfP5 subfraction. (A) Representative full-scan MS spectrum showing the major precursor ions detected in SfP5, including the prominent signal at *m*/*z* 403. The red and green boxes highlight the precursor ions at *m*/*z* 304 and 330 selected for MS/MS analysis. (B) MS/MS fragmentation spectrum of the precursor ion at *m*/*z* 304. (C) MS fragmentation spectrum of the precursor ion at *m*/*z* 330. (D) MS/MS fragmentation spectrum of the precursor ion at *m*/*z* 330. Fragmentation of the *m*/*z* 330 precursor yielded the *m*/*z* 304 ion, and both precursors generated closely related product-ion spectra dominated by fragments at *m*/*z* 182 and 150, suggesting that these ions correspond to structurally related molecular species.
