# Supplementary material for: Enhancing Neuronal Networks with Rhinella schneideri Skin Secretion Molecules: Implications for Neurodegenerative Disorders
Source: Toxins (Basel). 2026 Jun 20;18(6):271. doi: 10.3390/toxins18060271 (PMC13308062; doi:10.3390/toxins18060271)
Supplement: Supplementary file 1 [file toxins-18-00271-s001.zip › toxins-4233074-supplementary.pdf]

Supplementary Materials: Enhancing Neuronal Networks with *Rhinella schneideri* Skin Secretion Molecules: Implications for Neurodegenerative Disorders

Giovanna Arruda Caires, Isabela Souza Pereira, Carlos DeOcesano-Pereira, Daniel Carvalho Pimenta, Irina Kerkis, Juliana Mozer Sciani and Hugo Vigerelli

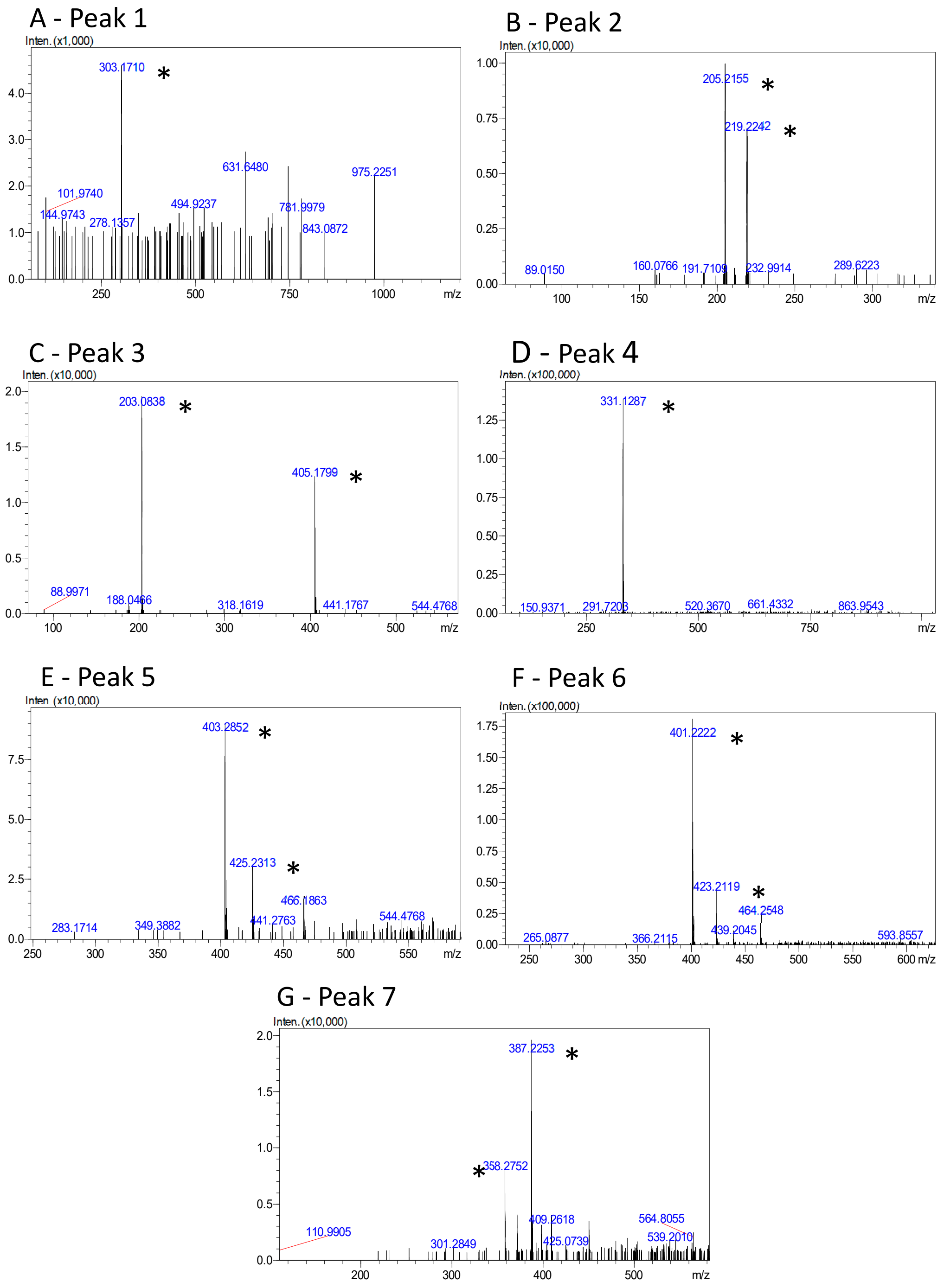

Figure S1. The numbers indicated above the peaks correspond to the compounds detailed in Table 1. Mass spectrometry analysis of the crude skin secretion (SS). Representative mass spectrometry (MS) spectra of the major peaks collected from the crude skin secretion of *Rhinella schneideri*. The numbers indicated above the signals (Peak 1, Peak 2, etc.) correspond to the specific peaks and their respective putatively identified compounds previously detailed in Table 1.

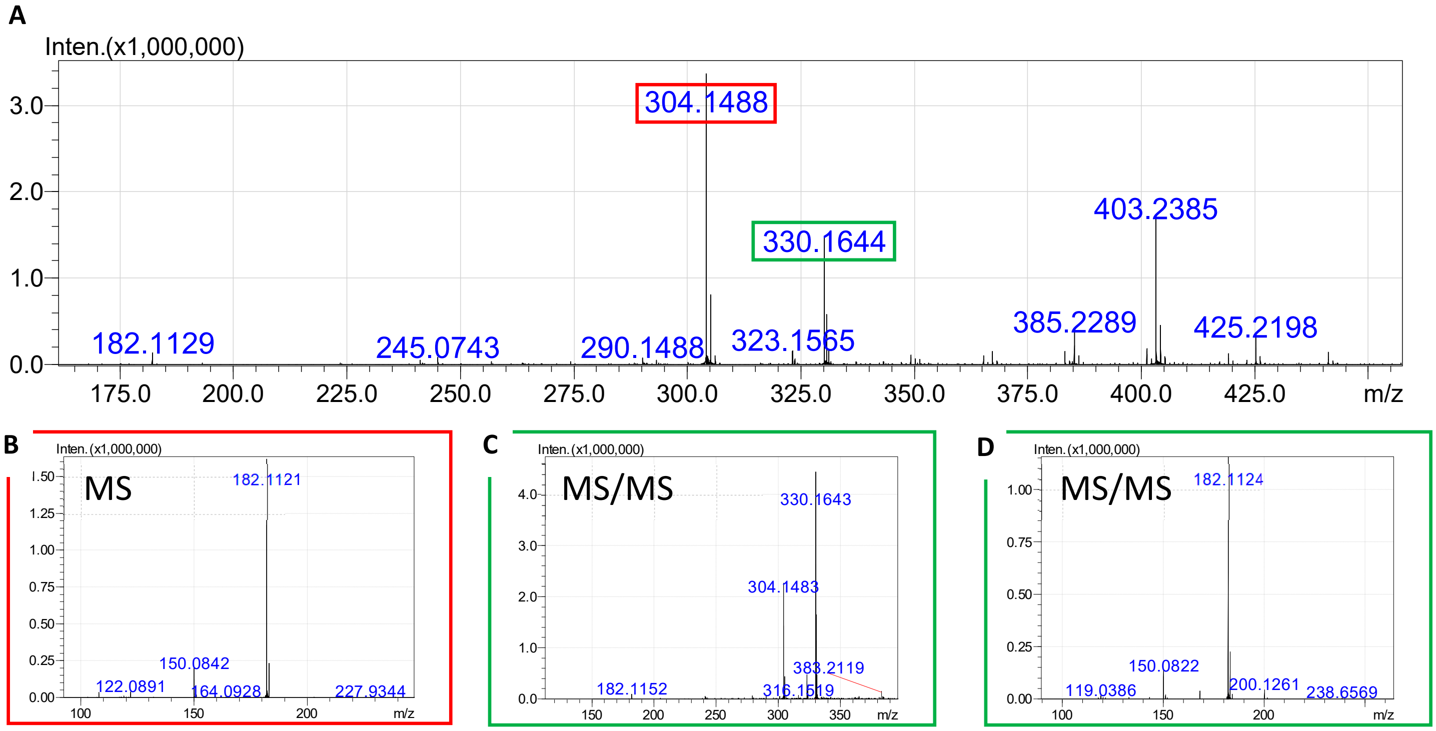

Figure S2. Mass spectrometry (MS) and tandem mass spectrometry (MS/MS) characterization of the biologically active SfP5 subfraction. (A) Representative full-scan MS spectrum showing the major precursor ions detected in SfP5, including the prominent signal at m/z 403. The red and green boxes highlight the precursor ions at m/z 304 and 330 selected for MS/MS analysis. (B) MS/MS fragmentation spectrum of the precursor ion at m/z 304. (C) MS fragmentation spectrum of the precursor ion at m/z 330 (D) MS/MS fragmentation spectrum of the precursor ion at m/z 330. Fragmentation of the m/z 330 precursor yielded the m/z 304 ion, and both precursors generated closely related product-ion spectra dominated by fragments at m/z 182 and 150, suggesting that these ions correspond to structurally related molecular species.
